# Pyogenic arthritis of native joints due to *Bacteroides fragilis*: Case report and review of the literature

**DOI:** 10.1097/MD.0000000000003962

**Published:** 2016-06-24

**Authors:** Joan M. Nolla, Oscar Murillo, Javier Narvaez, Carmen Gómez Vaquero, Jaime Lora-Tamayo, Salvador Pedrero, Javier Cabo, Javier Ariza

**Affiliations:** a Rheumatology Department, IDIBELL-Hospital Universitari de Bellvitge, Barcelona, Spain; b Infectious Diseases Department, IDIBELL-Hospital Universitari de Bellvitge, Barcelona, Spain; c Orthopedic Surgery Department, IDIBELL-Hospital Universitari de Bellvitge, Barcelona, Spain.

**Keywords:** anaerobe, *Bacteroides fragilis*, infectious arthritis, pyogenic arthritis, rheumatoid arthritis

## Abstract

Pyogenic arthritis of native joints due to *Bacteroides fragilis* seems to be an infrequent disease. We analyzed the cases diagnosed in a tertiary hospital during a 22-year period and reviewed the literature to summarize the experience with this infectious entity.

In our institution, of 308 patients with pyogenic arthritis of native joints, *B fragilis* was the causative organism in 2 (0.6%) cases. A MEDLINE search (1981–2015) identified 19 additional cases.

Of the 21 patients available for review (13 men and 8 women, with a mean age, of 54.4 ± 17 years), 19 (90%) presented a systemic predisposing factor for infection; the most common associated illness was rheumatoid arthritis (8 patients). Bacteremia was documented in 65% (13/20) of cases. In 5 patients (24%), 1 or more concomitant infectious process was found. Metronidazole was the most frequently used antibiotic. Surgical drainage was performed in 11 cases (52%). The overall mortality rate was 5%.

Pyogenic arthritis of native joints due to *B fragilis* is an infrequent disease that mainly affects elderly patients with underlying medical illnesses and in whom bacteremia and the presence of a concomitant infectious process are frequent conditions.

## 1 Introduction

Pyogenic arthritis presents 2 different scenarios^[1]^ depending on whether the infection compromises prosthetic or native joints. The 2 processes have important distinctive clinical and microbiological features and require different approaches.

Pyogenic arthritis of native joints^[2]^ is a potentially life-threatening disease that can lead to rapid joint destruction and irreversible loss of function. It remains a serious medical emergency with high morbidity and mortality. A large number of gram-positive and gram-negative bacteria have been identified as causative agents.^[3,4]^ Overall, *Stahylococcus aureus* has been the most commonly isolated microorganism, accounting for more than 50% of reported cases; streptococci cause almost 25% of cases and gram-negative bacilli around 10% to 15%. Anaerobic etiological agents are extremely rare; in a recent review^[3]^ of 3-decade trends in the distribution of organisms causing septic arthritis in native joints, anaerobic bacteria caused only 2 of the 374 cases reported.

*Bacteroides fragilis* is an obligate anaerobe, a gram-negative rod that forms a part of the normal flora of the oral cavity, the skin, and the genitourinary and gastrointestinal tract. Like other microorganisms of the genre *Fusobacterium, Porphyromonas, Prevotella*, and *Peptostreptococcus* it is a nonspore forming bacteria.

*B fragilis* can become an opportunistic pathogen. In fact, it is the most commonly isolated organism in anaerobic infections and responsible for a wide spectrum of clinical manifestations. The commonest entities^[5]^ are primary bacteremia, periodontal disease, skin and soft tissue infections, and intra-abdominal abscesses. Other relevant but less frequent conditions are respiratory tract infections,^[6]^ endocarditis,^[7]^ pericarditis,^[8]^ meningitis,^[9]^ and osteoarticular infections.^[10–12]^

The prevalence of *B fragilis* arthritis of native joints has not been established, but it seems to be an extremely rare disease. At the modern era, Ziment et al^[13]^ performed the first detailed description of a case in 1969. Additionally, 4 new cases^[14–17]^ were reported in the following decade, demonstrating the capacity of this organism to cause pyogenic arthritis. In 1990, Rosenkranz et al^[18]^ presented their experience, reviewed the literature, and established the state of the art about this topic.

In the current study, we present the cases of pyogenic arthritis of native joints due to *B fragilis* observed at our institution over 2 decades, and review the available literature to summarize the experience with this infectious entity and to clarify, in the light of the current knowledge, certain clinical, and therapeutic issues of the disease.

## 2 Patients and methods

We searched the database of our hospital (a 700-bed tertiary care teaching institution in Barcelona that does not treat pediatric, obstetric, or burn patients) for subjects with infectious arthritis admitted from January 1992 to December 2013.

For the present study, we excluded: patients with prosthetic joint infection, patients with postoperative arthritis (patients who had undergone joint surgery or arthroscopy in the year before diagnosis), patients with arthritis secondary to traumatic or spontaneous skin or soft tissue ulcers (e.g., diabetic foot), and patients with mycobacterial, brucellar, or fungal arthritis.

Three hundred eight patients [203 male (66%), 105 (34%) female; mean age: 57.7 ± 16.3 years] with pyogenic arthritis of native joints were identified. All cases were microbiologically proven. According to our protocol, joint fluid samples are sent directly to the laboratory, processed in liquid (thioglicolate) and solid media (5% sheep blood, chocolate, and MacConkey agar), and incubated for at least 7 days. Blood samples are cultured following standard recommendations in bottles of BacT (Bactec NR-860-system, Johnson Laboratories, Towson, MD) with both aerobic and anaerobic media. Microorganisms and their antibiotic susceptibility are identified using the MicroScan system (Dad Behring, West Sacramento, CA).

Two patients with *B fragilis* arthritis were observed at our center during the assessment period.

Previously published cases of *B fragilis* pyogenic arthritis of native joints were identified using a computerized search of the MEDLINE (National Library of Medicine, Bethseda, MD) database from January 1981 to December 2015. The key words used were “*Bacteroides fragilis”* and “arthritis.” Only reports in English, French, and Spanish were considered; the references studies obtained were then examined to identify additional reports. Only cases that were sufficiently detailed to be analyzed individually were included.

We excluded: patients under the age of 18, cases of prosthetic joint infection^[18–21]^; and cases of postoperative arthritis. A case^[22]^ of polymicrobial arthritis involving *B fragilis* was not included.

In accordance with the guidelines of our institutional ethics committee, formal approval for this study was not required. Informed consent was not obtained from the patients, but their clinical records and information were anonymized before analysis.

## 3 Results

Nineteen cases of pyogenic arthritis of native joints due to *B fragilis* were identified in the literature.^[18,23–38]^ Thus, including the 2 patients observed at our center during the study period, 21 cases were available for review (Table 1  ).

**Table 1:**
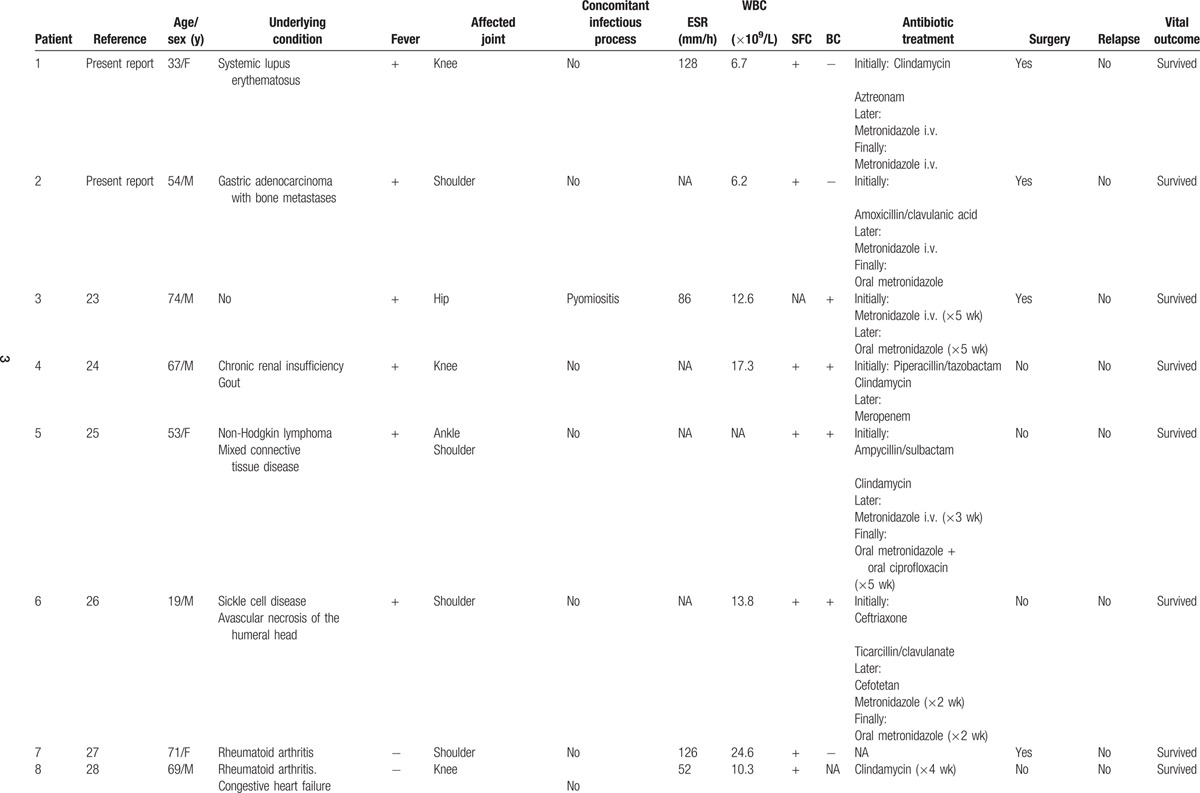
Demographic, clinical, and laboratory findings, treatment and outcome in 21 cases of pyogenic arthritis of native joints due to *Bacteroides fragilis*.

**Table 1 (Continued):**
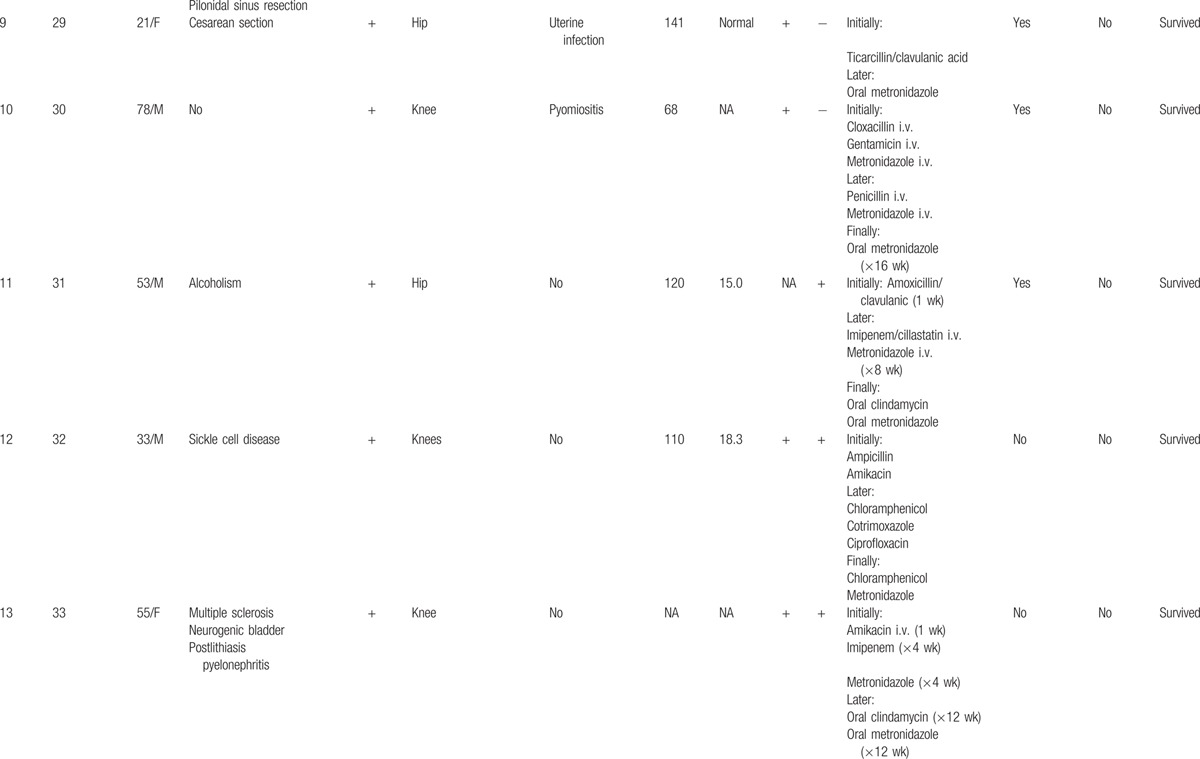
Demographic, clinical, and laboratory findings, treatment and outcome in 21 cases of pyogenic arthritis of native joints due to *Bacteroides fragilis*.

**Table 1 (Continued):**
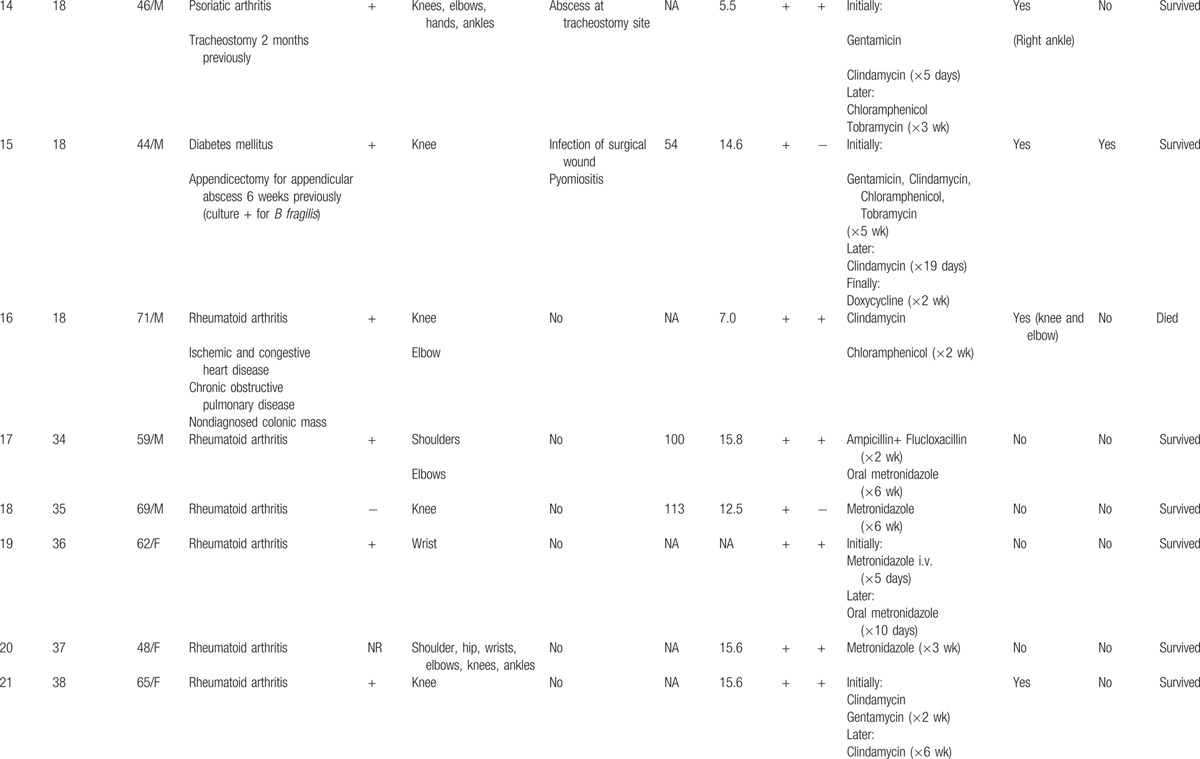
Demographic, clinical, and laboratory findings, treatment and outcome in 21 cases of pyogenic arthritis of native joints due to *Bacteroides fragilis*.

Of these 21 patients, 13 (62 %) were men and 8 (38 %) women, with ages ranging from 19 to 78 years (54.4 ± 17 years). Nine patients (43%) were over 60 years of age.

Nineteen patients (90%) presented a systemic predisposing factor for infection. The most common associated illness was rheumatoid arthritis (8 patients). Other relevant but more infrequent conditions were inflammatory diseases (systemic lupus erythematosus, mixed connective tissue disease, and psoriatic arthritis, 1 case each) and cancer (1 case of gastric adenocarcinoma with bone metastases and 1 case of non-Hodgkin lymphoma).

Fifteen percent (3/20) of patients did not present with fever before diagnosis. Fifteen patients (71%) presented monoarthritis. The knee was the most frequently compromised joint (12 patients).

The erythrocyte sedimentation rate (ESR) was above 50 mm/h in all cases in whom this datum was reported. Mean ESR was 99.8 ± 30.7 (range: 52–128). In 72% of patients (10/14) the white blood cell count (WBC) was above 11 × 10^9^/L; the mean WBC was 13.2 ± 5.1 × 10^9^/L (range, 5.5–24.6). Synovial fluid cultures gave positive results in all cases in whom they were performed (20/21). Blood cultures gave positive results in the 65% of cases (13/20).

Five patients (24%) presented 1 or more concomitant infectious processes due to the same microorganism. The most frequent was pyomiositis (3 patients); other entities were uterine infection, abscess after tracheostomy, and surgical wound infection.

Initial empirical therapy varied among patients, whereas metronidazole was the most used antibiotic after bacterial identification. As part of the standard management, some patients underwent daily percutaneous articular drainage. Surgical debridement was performed in 11 (52%) patients.

One patient (5%) presented relapse and 1 (5%) died.

## 4 Discussion

Although Pasteur in one of his earliest publications described obligatory anaerobic bacteria, anaerobic infection other than that caused by certain species of clostridia received relatively little attention over the following 100 years. In 1974, Gorbach and Bartlett^[39–41]^ reviewed the available literature on pyogenic processes associated with anaerobic organisms of the normal flora and provided your expertise. These authors stated that anaerobic bacteria are rarely encountered in pyogenic arthritis.

It is established that anaerobic arthritis mainly affects patients undergoing surgical treatment for traumatic injuries or elective musculoskeletal surgery (arthroplasty or other orthopedic devices); when it occurs, *B fragilis* is the principal causative microorganism.^[11]^ However, information on anaerobic arthritis of native joints is very scarce.

In the present study, we reviewed all cases of *B fragilis* pyogenic arthritis of native joints published in the literature since 1981 and included 2 patients attended at our hospital. The summarized experience, comprising 21 cases, is, by far, the largest series on this infectious disease.

Data from our institution confirm that *B fragilis* is an exceptional causative agent of pyogenic arthritis of native joints, representing only 0.6% of all diagnosed cases. The figure obtained for overall anaerobic infection is similar to than observed in the series of adult patients with infectious arthritis published in the literature over the last decade,^[3,42–47]^ with the exception of the series of Lim et al^[42]^ who reported an high frequency of patients with prosthetic joint infection (21%). Nevertheless, it is possible that the failure to culture under anaerobic conditions will underestimate the real importance of *B fragilis* as a causative agent.

Our review demonstrates that *B fragilis* arthritis mainly appears in elderly patients. A predominance among males was also noted as also occurs in the general series of pyogenic arthritis.^[3,42–47]^

The presence of concomitant chronic debilitating disease in the vast majority of patients (90%) indicates the opportunistic nature of the microorganism. Ischemic heart disease, sickle cell disease, malignancy, and above all rheumatoid arthritis (40%) were the main underlying processes.

The association between *B fragilis* pyogenic arthritis and rheumatoid arthritis has been previously reported^[18]^ and should be particularly emphasized according to the data included in the present revision.

Overall, patients with rheumatoid arthritis are particularly susceptible to pyogenic arthritis. The estimated incidence of this complication ranges from 0.3% to 3%.^[48]^*S aureus* is the main etiological agent, causing more than 70% of cases. The frequency of anaerobic arthritis is low, though it seems higher than observed in the general population. In the classical review of Gardner and Weisman,^[49]^ anaerobes accounted for 3% of cases (6/213) observed between 1946 and 1987. In our experience,^[4,27,50]^ anaerobic bacteria were the causative agents of 6% of cases (2/35) attended between 1981 and 2013. The mechanisms responsible for the increased vulnerability to joint infection have not been precisely identified, but skin defects, previous articular damage, poor clearance of bacteria from the joint, and acquired phagocytic defects secondary to drugs or disease have been proposed.^[48,49]^ In the particular case of *B fragilis* additionally is important to consider the relevant role of its capsula as a virulent factor,^[51]^ promoting abscess formation by inhibiting opsonophagocytosis. Thus, the damaged joint along with the systemic immunologic impairment may be the key factors in the development of arthritis by *B fragilis* in patients with rheumatoid arthritis.

The percentage of patients who remained afebrile before diagnosis was low, but noticeable. These cases stress the importance of maintaining a high index of suspicion of infection when 1 joint (or more) suddenly becomes inflamed, especially in immunocompromised hosts. The infection involved more than 1 joint in almost 30% of cases, whereas the classically reported frequency of polyarthritis is 15%.^[52]^ As in the general series of pyogenic arthritis,^[3,4,42–47]^ the knee was the most commonly affected joint.

The high percentage of cases with positive blood cultures should also be noted; bacteremia was detected in 65% of the cases in whom blood culture results were available. Most cases of *B fragilis* arthritis have been attributed to hematogenous spread from a distant infected focus, usually intra-abdominal. However, on occasions this focus has not been identified despite a search strategy that includes colonoscopy and computerized tomography of the abdomen.^[24]^

In our review, a quarter of patients present a concomitant infectious process, mainly pyomiositis as a result of contiguous spread of the infection. This circumstance must be borne in mind since the symptoms and signs of pyogenic arthritis and pyomiositis are similar and one of them may be missed or incompletely treated.^[23]^

Anaerobic and aerobic arthritis require the same treatment,^[53]^ comprising temporary immobilization, drainage of the joint, and adequate antibiotic therapy. Some authors^[31]^ favor hyperbaric oxygen therapy as an adjunctive treatment in the management of the patients if the preservation or restoration of the blood supply in the area involved is not possible.

Prompt complete removal of infected synovial fluid is necessary to preserve good joint function and to control the infection. This aids bacterial clearance from synovial fluid and tissues, rapidly decreases the intra-articular pressure, reduces cartilage damage, and increases the efficacy of antimicrobial therapy. No large prospective comparative studies are available, and the choice of drainage (repeated closed needle aspiration or surgical drainage) remains controversial.^[54]^

Few data are available on the antibiotic therapy of arthritis due to *B fragilis* and no recommendations have been clearly established. For treatment of anaerobic bacterial infections, clindamycin has classically considered the gold standard. However, *B fragilis* antibiotic resistance to clindamycin has increased over the years, reaching 41.8% in Mediterranean countries^[55]^ and now, empirical therapy by this antibiotic should be discouraged.^[12]^

Data from this review show that initial empirical therapy varied among patients, while metronidazole was the most used antibiotic after bacterial identification. While β-lactam antibiotics resistant to β-lactamases may be considered good alternative therapy if an appropriate articular drainage is warranted, metronidazole provides a better theoretical profile because its reported bactericidal activity in the purulent milieu of the arthritis.^[56,57]^

Relapse seems to be rare in *B fragilis* arthritis, being observed in only 1 of the 21 cases analyzed. The mortality rate (5%) was consistent with recently published reports^[3,4]^ of native pyogenic arthritis. Underlying medical processes and bacteremia are the main determinants of death in patients with infectious arthritis.^[4]^ The difficulty of modifying these variables means that it is hard to reduce mortality rates despite advances in the medical and surgical management of these patients.

In summary, *B fragilis* is an infrequent causative agent of pyogenic arthritis of native joints. Joint disease due to this microorganism mainly affects aged patients with underlying medical illnesses, in whom bacteremia and the presence of a concomitant infectious process are frequent conditions. Relapse seems to be uncommon and the mortality rates are consistent with those reported in native pyogenic arthritis.
